# Ferulic acid as a therapeutic agent in depression: Evidence from preclinical studies

**DOI:** 10.1111/cns.14265

**Published:** 2023-05-14

**Authors:** Xiaoyu Dong, Dongxue Zhao

**Affiliations:** ^1^ Department of Neurology Shengjing Hospital of China Medical University Shenyang Liaoning China

**Keywords:** animal models, depression, ferulic acid, neuroinflammation, pharmacology, systematic review

## Abstract

Depression is a common but severe mood disorder with a very high prevalence across the general population. Depression is of global concern and poses a threat to human physical and mental health. Ferulic acid (FA) is a natural active ingredient that has antioxidative, anti‐inflammatory, and free radical scavenging properties. Furthermore, studies have shown that FA can exert antidepressant effects through a variety of mechanisms. The aim of the review was to comprehensively elucidate the mechanisms in FA that alleviate depression using animal models. The in vivo (animal) studies on the mechanism of FA treatment of depression were searched in PubMed, Chinese National Knowledge Infrastructure, Baidu academic, and Wan fang databases. Thereafter, the literature conclusions were summarized accordingly. Ferulic acid was found to significantly improve the depressive‐like behaviors of animal models, suggesting that FA is a potential natural product in the treatment of depression. The mechanisms are achieved by enhancing monoamine oxidase A (MOA) activity, inhibiting microglia activation and inflammatory factor release, anti‐oxidative stress, promoting hippocampal nerve regeneration, increasing brain‐derived neurotrophic factor secretion, regulating gut microbiome, and activating protein kinase B/collapsin response mediator protein 2 (AKT/CRMP2) signaling pathway. Ferulic acid produces significant antidepressant effects in animal depression models through various mechanisms, suggesting its potential value as a treatment of depression. However, clinical research trials involving FA are required further to provide a solid foundation for its clinical application.

**Significance statement**

Ferulic acid (FA) is a phenolic acid extracted from the resin of Asafetida. Asafetida is a perennial herb of the Umbelliferae family. Ferulic acid is a common aromatic acid in the plant kingdom. It is a component of suberin. It rarely exists in a free state in plants and mainly forms a combined state with oligosaccharides, polyamines, lipids, and polysaccharides. It has many healthcare functions, such as scavenging free radicals, antithrombosis, antibacterial and anti‐inflammatory, inhibiting tumors, preventing and treating hypertension, heart disease, and enhancing sperm motility. Ferulic acid has low toxicity and is easy to be metabolized by the human body. It can be used as a food preservative and has a wide range of uses in food and medicine.

This review aims to explore the mechanism by which ferulic acid alleviates depression in animal depression models (in vivo), provide a useful experimental basis for clinical research, and explore ideas for the development of new antidepressants. In a systematic review of previous studies, we find that FA exerts important neuroprotective effects in CNS diseases through antioxidant, anti‐inflammatory, anti‐apoptosis, and other mechanisms. Moreover, FA was found to alleviate abnormal depressive behaviors in multiple animal depression models, underscoring the potential use of FA as a new antidepressant agent. Given the lack of knowledge explaining the antidepressant mechanism of FA, we believe that our study makes a significant contribution to the literature because prior studies confirm FAs' impact on treating dementia, Parkinson's disease, cardiovascular disease, diabetes, cirrhosis, and depression.

## 1 INTRODUCTION

Depression is a severe mental illness with consequences for human physical and mental health.^1^ Clinically, depression is characterized by depressed moods, impaired thinking, and volitional inactivity.^2^ The annual prevalence of major depressive disorders in North America is 4.5%, and the incidence of depression is higher in less developed areas. The World Health Organization (WHO) predicts that depression will become the global leading cause of disability within the next 2 years.^3^ Furthermore, depression is projected to become the largest burden worldwide by 2030.^4^ The pathogenesis of depression is still controversial, and is considered to result from the combination of genetic, environmental, psychological, and biological factors.^5^ Several widely accepted theories for its development include reduced serum serotonin (5‐HT) levels, reduction in brain‐derived neurotrophic factor (BDNF) release, hyperfunction of hypothalamic pituitary adrenal (HPA), and inflammatory responses.^6^, ^7^, ^8^ Given the gradual increase of in‐depth depression studies, it has been found that mitochondrial metabolic disorders also contribute to the pathogenesis of depression.^9^, ^10^ Despite the significant progress made in the treatment of depression over the past few decades, most clinically developed antidepressant drugs have been associated with unsatisfactory therapeutic effects. Furthermore, these drugs generally have long treatment cycles, severe side effects, and an increased risk of drug dependence.^11^ Therefore, the development of highly efficient antidepressant drugs with minimal side effects has become pertinent to the treatment of depression. According to the WHO, approximately 80% of the global population still relies on plant medicines.^12^ Phytomedicines are significant gifts from nature. Many natural products that have been isolated from phytomedicines, such as paclitaxel, artemisinin, ginkgolide B, and curcumin, have been structurally modified to yield great medical value.^13^ Therefore, it is important to investigate natural products that can serve as potential drugs in the treatment of depression.

**FIGURE 1:**
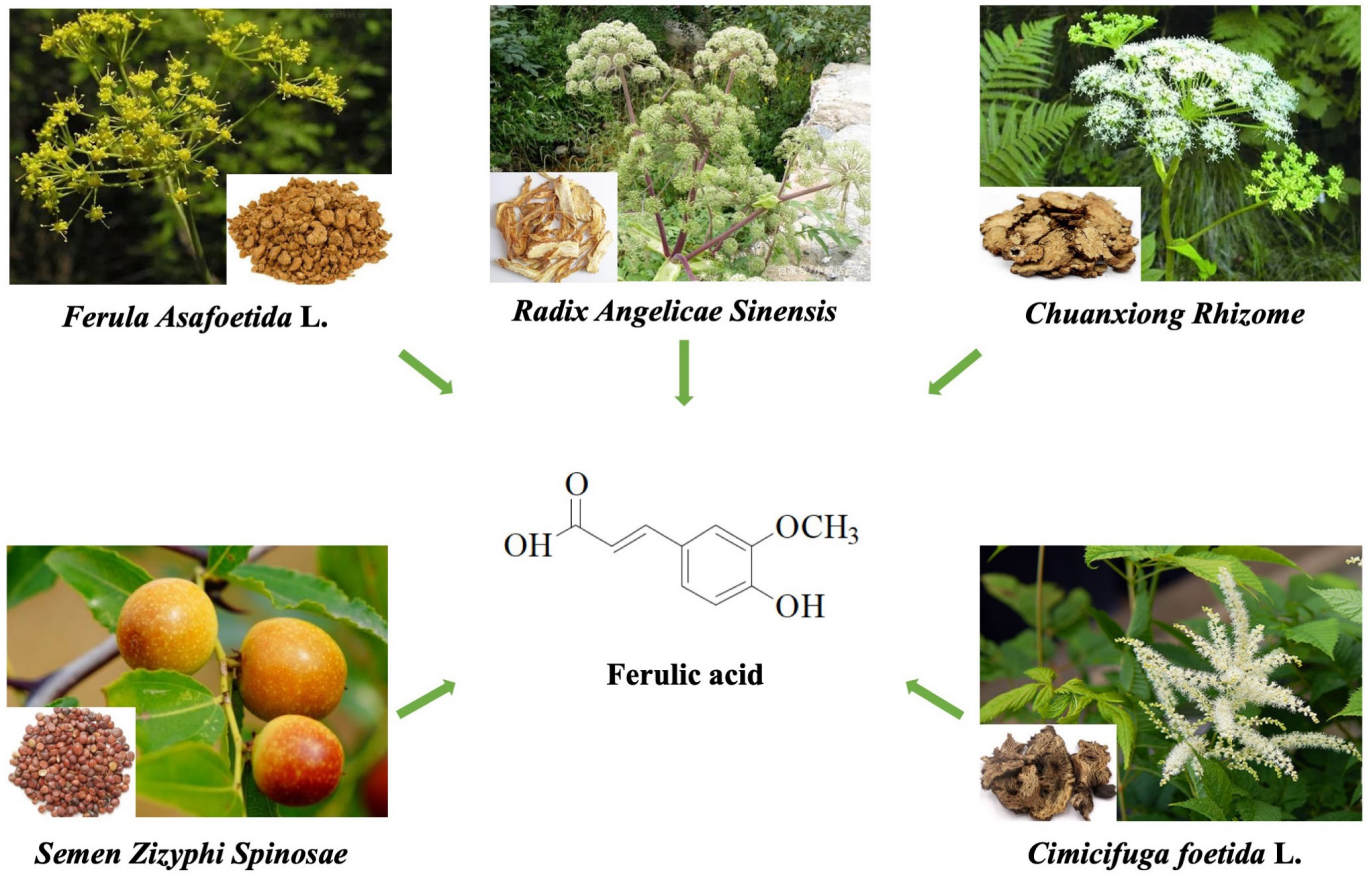
Ferulic acid and the main source of Chinese herbal medicines.

The chemical name of FA is 4‐hydroxy‐3‐methoxycinnamic acid, which is one of the derivatives of cinnamic acid (also known as cinnamic acid, 3‐phenyl‐2‐acrylic acid). Originally found in the seeds and leaves of plants, FA is a phenolic acid that is present widely in plants. Ferulic acid combines with polysaccharides and proteins to form the skeleton of the cell wall.^14^ A typical Mediterranean diet is rich in plant‐based foods (including breads, grains, vegetables, and fruits) and low to moderate in fish, red meat and wine, with daily FA intake of about 150–250 mg (16–24 mol/kg body weight).^15^ However, FA intakes are only theoretical (Table 1), as they vary according to dietary habits and the number of vegetables/fruits per day.^16^, ^17^, ^18^, ^19^ Furthermore, FA is prevalent in Chinese herbal medicines (CHMs), found in plants such as *Ferula Asafetida* L., *Radix Angelicae Sinensis*, *Chuanxiong Rhizome*, *Cimicifuga foetida* L., and *Semen Zizyphi Spinosae* ^20^, ^21^ (Figure 1). Ferulic acid is an active ingredient of these CHMs and has been used as one of the quality indicators of CHMs. Currently, significant evidence indicates that FA has various functions such as anti‐oxidation,^22^ anti‐apoptosis,^23^ inhibition of neuroinflammation,^24^ scavenging free radicals,^25^ and promoting nerve regeneration.^26^ Therefore, FA can be used to treat a variety of diseases, including dementia, Parkinson's disease,^27^ cardiovascular disease,^28^ diabetes,^29^ and cirrhosis.^30^

**TABLE 1:** Approximate amounts of ferulic acid in some foods.

Types of food	FA (mg/100 g)	Average daily portion (g/day)	Ingested amount of FA (mg/day)
Cereal brans	1351–3300	5 (20)	68–165 (270–660)
Popcorn	313	60	187.8
Artichokes	275	250	688
Coffee	9.1–14.3	200	18.2–28.6
Eggplants	7.3–35	200 (250)	15–70 (18–87)
Pasta	12	100 (80)	12 (9.6)
Grapefruit	11	125 (150)	13.75–16.5
White wheat bread	8.2	35 (50)	2.87 (4.10)
Orange	9.5	125 (150)	11.88–14.25
Tomatoes	6	200 (250)	12 (15)
Banana	5.4	125 (150)	6.75–8.1
Broccoli	4.1	200 (250)	8.2 (10.25)

Abbreviation: FA, ferulic acid.

Notably, FA has shown significant results in antidepression.^31^ However, research that systematically explains the antidepressant mechanism of FA is limited. Therefore, this review aims to explore the mechanism of FA that alleviates depression using animal depression models (in vivo). Furthermore, this review aims to provide a useful experimental basis for clinical research and explore innovative ideas for the development of new antidepressants.

## 2 FA: PHYSICOCHEMICAL PROPERTIES AND BIOSYNTHESIS

The molecular formula of FA is C10H10O4, with a molecular weight of 194.18, and a melting point of 169–173°C.^32^ Ferulic acid is highly soluble in methanol, ethanol, and acetone. It is soluble in hot water, slightly soluble in cold water and petroleum ether, and insoluble in benzene. Furthermore, FA can form salt compounds with metal ions to obtain good pH stability. The molecular structure of FA can be divided into two types: cis‐form and trans‐form. The cis‐form, generally, is a yellow oily substance, whereas the trans‐form is a white to yellowish square or fibrous crystals. Ferulic acid has strong antioxidant and reduction properties and is easily decomposed when exposed to light.^33^

Ferulic acid can be obtained from plants in three ways: first, from binding with certain small molecules; second, from extraction from its plant cell wall; third, extraction from tissue culture. Ferulic acid in plants is crosslinked usually with polysaccharides and lignin through ester bonds or self‐esterification or etherification to form di‐FA. Ferulic acid can be released by breaking ester bonds by alkali and enzymatic methods, and then extracted with appropriate solvents.^34^, ^35^

The chemical synthesis method of FA uses vanillin as the basic raw material and Wittig–Horner reaction (WHR) and Knoevenagel reaction (KR) as the main organic reactions. During the first method, FA is synthesized with WHR. The WHR of triethyl phosphite acetate and acetyl vanillin combine in a strong base system followed by acidification with concentrated hydrochloric acid to obtain FA. The method needs to first protect phenolic hydroxyl groups in advance. Due to the presence of a strong base, the formation of sodium phenol will inhibit the reaction between the carbonyl group and carbanion, which is also prone to side reactions that generate impurities. For the second method, FA is synthesized with KR. A KR between vanillin and malonic acid was conducted to produce ferulic acid by adding a small amount of organic base in pyridine solvent as a catalyst. The catalysts include piperidine and aniline. However, the method has a long reaction time of up to 3 weeks, obtained from a mixture of trans‐FA and cis‐FA.^36^ In addition, several microorganisms can be used to convert FA precursors to FA by biosynthesis. For example, the butanol cinnamate ester extracted from clove oil can be converted to FA.^37^, ^38^ Even though biosynthesis is a clean and effective synthesis method, it is yet to be mass‐produced.

## 3 SEVERAL PATHOGENESES INVOLVED IN DEPRESSION

The pathogenesis of depression is complex and involves the biological, physiological, and social environment. The monoamine hypothesis was proposed in the 20th century and prompted revolutionary progress in the treatment of depression.^39^ Most clinical studies have indicated that the onset of depression is mainly due to reduced concentrations of monoamine neurotransmitters such as 5‐HT and dopamine (DA).^40^ This may be related to the increased activity of indoleamine 2, 3‐dioxygenase, and the accelerated decomposition rate of tryptophan among patients with depression, thus inhibiting the metabolism of tryptophan to the 5‐HT pathway.^41^ In addition, studies have shown that the concentration of dopamine transporter (DAT) is significantly higher among patients with depression compared with the general population. Furthermore, the high concentration of DAT in the body can increase the recovery of DA at the synaptic terminal, which is manifested as a decrease in DA levels in the synaptic space, ultimately inducing depression.^42^, ^43^

Hypothalamic pituitary adrenal is an endocrine axis that maintains homeostasis and stress responses. Furthermore, HPA occupies a unique role in the onset and development of depression.^44^ It is clinically found that the hyperactivity associated with the HPA axis in patients with depression manifests mainly due to increased levels of hormones (such as corticotropin‐releasing hormone [CRH] and glucocorticoid [GC]) in the body.^45^ In rats treated with GC, it was found that CRH‐positive neurons in the paraventricular nucleus of the hypothalamus, adrenocorticotropic hormone (ACTH)‐positive neurons in the anterior pituitary, and CRH‐positive nerve fibers in the median eminence were reduced significantly, resulting in depressive behavior.^46^ Furthermore, the hyperfunction of the HPA axis promotes the secretion of excessive GC, which can damage the hippocampal neurons.^47^ The damaged hippocampus cannot inhibit the abnormal activity of HPA function effectively, thus forming a vicious circle that can eventually induce depression and cause cognitive‐related dysfunction.

The onset of depression is closely related to the inflammatory response of the body.^48^ Excessive pro‐inflammatory factors can reduce the utilization rate of tryptophan precursor and C‐reactive protein, which are closely related to 5‐HT synthesis. In turn, this can reduce the concentration of neurotransmitters, ultimately causing depression.^49^ Furthermore, the overexpression of inflammatory factors can increase the secretion of hypothalamic regulatory hormones, which leads to the overactivation of the HPA axis, exacerbating the depression‐like behavior among the rats. Thus, this suggests that inflammatory responses could be an important mechanism of depression.^50^

Neurotrophic factor (NTF) plays an important role in the occurrence, development, and functional maintenance of the central nervous system (CNS).^51^ Neurotrophic factor can provide nutritional support for neurons and pertinent brain regions in the CNS related to the regulation of emotional behavior.^52^ Brain‐derived neurotrophic factor is a common NTF that uses physiological roles to change synaptic plasticity and increase synaptic connections.^53^ Studies have shown that the content of BDNF in the hippocampus and prefrontal cortex of patients with depression is significantly reduced, whereas the level of BDNF is significantly increased after antidepressant intervention in patients with depression.^54^ In addition to the BDNF, a series of cascade reactions caused by BDNF binding to the level of its receptor play an important role in the pathogenesis of depression and antidepressant intervention. For example, BDNF can bind to Tyrosine kinase receptor B (Trk B) to activate intracellular phosphatidylinositol‐3‐hydroxykinase (PI3K), mitogen‐activated protein kinase (MAPK), and phospholipase C (PLC) signaling pathways, contributing to physiological roles, such as regulating synaptic plasticity, increasing the expression of some monoamine neurotransmitter receptors, and promoting the repair and regeneration of neurons.^55^, ^56^

Evidence has shown that hippocampal neurogenesis plays an important role in depression.^57^ Disruption of hippocampal neurogenesis and blockage of neurogenesis using focal radiation can induce depression‐like behavior and result in a reduced antidepressant treatment effect. This suggests that neurogenesis is involved in the onset of depression and resembles the therapeutic effects of antidepressants to a certain extent.^58^ Mitochondria have potential roles in the complex processes of producing adenosine triphosphate (ATP), establishing membrane stability, signaling intracellular Ca^2+^, reactive oxygen species (ROS) homeostasis, and performing neurotransmission and plasticity.^9^ Studies have also shown that glucose utilization decreased in the prefrontal cortex, anterior cingulate gyrus, and caudate nucleus of patients with depression.^59^, ^60^ This suggests that depression may be caused by the impairment of brain energy owing to mitochondrial dysfunction.^61^, ^62^

In recent years, the relationship between gut microbiota and diseases has attracted more and more attention. Studies have shown that the imbalance of gut microbiota is closely related to the occurrence of depression. Gut microbiota can affect the host's behavior and mood through the microbiota–gut–brain axis, and then induce depressive symptoms.^63^ Compared with healthy adults, patients with depression have different gut microbiota diversity, microbial richness and evenness, and a smaller number of bacterial taxa are associated with depression.^64^ The proportion of *Actinobacteria* in the intestine of patients with depression increased,^65^ and the abundance of *Bacteroides* decreased.^66^ In addition, the abundance of *Prevotella* in the gut of patients with depression is low, and depressive symptoms are also negatively correlated with *Prevotella*.^65^ Moreover, *Actinobacteriaceae*, *Cocoanicaceae*, *Bifidobacteriaceae*, *Lactobacillaceae*, *Pyrospirillaceae*, and *Streptococcaceae* are highly abundant in depression.^67^ Depression can be alleviated by regulating gut microbiota. Probiotic supplementation can significantly reduce the ratio of kynurenine to tryptophan in serum, restore the status of intestinal flora, inhibit the immune inflammatory cascade in the process of depression, and improve the related symptoms of patients with mental disorders through the changes in the distribution of intestinal flora and the regulation of intestinal permeability.^68^, ^69^

Traditional Chinese medicine (TCM) has made significant progress in the treatment of depression by delaying the onset and course of the illness, thus improving the efficacy of western medicine and reducing its side effects.^70^ Currently, TCM for the treatment of depression is derived mostly from Chinese medicine and active ingredients. Ferulic acid is a phenolic compound existing in a variety of CHMs, which may play a therapeutic role in depression through a variety of mechanisms. The subsequent section provides a systematic review of the antidepression effect of FA in animal models to gain an in‐depth understanding of the mechanism in FA that alleviates depression, providing a foundation for further application in clinical practice.

## 4 MATERIALS AND METHODS

### 4.1 Search strategy

The in vivo (animal) studies on the mechanism of FA treatment of depression were searched in PubMed, Chinese National Knowledge Infrastructure (CNKI), Baidu academic, and Wan fang databases. Studies published from the time of inception of each database to February 2022 for all English or Chinese languages were analyzed. The following expressions were used to search each database.

Ferulic acid
FA
or/(1)–(2)
Depression
Depressed
or/(4)–(5)
(3) and (6)

The initial screening of the studies was based on the title and abstract of the articles. Thereafter, the publications were reviewed in their entirety.

### 4.2 Inclusion and exclusion criteria

Studies on animal models of FA treatment of depression were included. These studies analyzed either the effect of FA on the depression model, FA's potential alleviating or therapeutic effect, or its mechanism through the in vivo behavior model. According to the statistical classification and writing requirements of this review, the eligibility criteria for inclusion in this systematic review were as follows:

1. All articles should be indexed and published in renowned journals with considerable impact factors.
2. Depression models should be treated with FA.
3. The studies must have a control group.
4. Research content must be associated with depression.

The exclusion criteria in this systematic review were as follows:

1. review articles;
2. books;
3. studies on hybrid preparations of FA and other drugs;
4. studies without a control group; and
5. in vitro studies.

### 4.3 Data extraction and treatment evaluation

Two authors independently collected the original literature and resolved disputes through discussion. The following information was retrieved from each study: (1) year of study publication and first author; (2) species used for in vivo studies; (3) model‐induced drugs with doses or induced experiments; (4) dosage and duration of FA treatment, and (5) main results and mechanisms of FA in treatment of depression.

## 5 RESULTS

**FIGURE 2:**
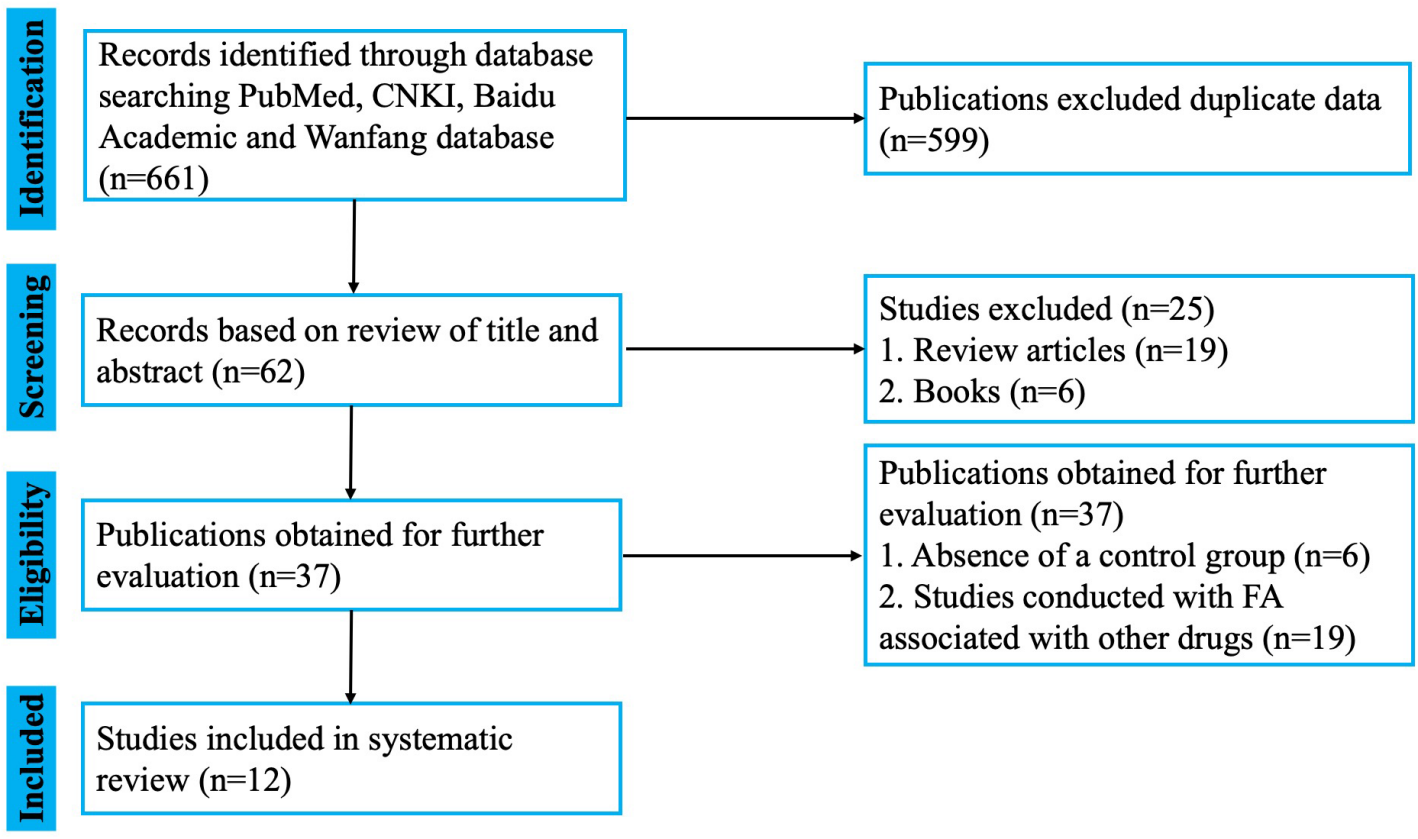
Prisma flowchart outlining the article screening process.

After the preliminary screening, 661 studies were included, of which 279 were found in PubMed, 198 in CNKI, 120 in Baidu Academic, and 64 in Wan fang databases. Due to duplication between databases, and after applying the exclusion and inclusion criteria, 37 studies were selected, and their abstracts were reviewed. Finally, 12 studies were included in this review (Figure 2).

### 5.1 Study characteristics

In this review, 12 in vivo studies were included. The following species were used: imprinting control region (ICR) mice (*n* = 7), Swiss albino mice (*n* = 3), Sprague–Dawley (SD) rats (*n* = 1), C56BL/6 mice (*n* = 1). Among the 12 studies, five studies utilized behavior test induced models, including forced swimming test (FST) and tail suspension test (TST); three studies used drugs to induce models: two studies utilized reserpine induced models, one study utilized corticosterone (CORT), and one study utilized lipopolysaccharide induced models. Another three studies utilized chronic stress‐induced models and one study utilized the inevitable stress‐induced model.

The amount of FA administered varied from 0.01 to 80 mg across the 12 studies over a duration ranging from 15 min to 4 weeks. Behavioral tests were used to observe the therapeutic effects, including the FST, TST, elevated plus‐maze test, sucrose preference test, splash test, thermal hyperalgesia, and mechanical allodynia. Additional data on these studies are provided in Tables 2 and 3.

**TABLE 2:** Studies that investigated the use of ferulic acid for depression.

Study (years)	Species (Sex, *n*)	Inducement	Interventions	Evaluated methods
Chen et al. (2015)	ICR mice (male, *n* = 10)	Unavoidable and inescapable stress	FA (10/20/40/80 mg/kg) for 30 min	FST, TST
Zhang et al. (2011)	SD rats (male, *n* = 10)	FST	FA (25/50 mg/kg) for 25 h	FST, OFT
Xu et al. (2013)	ICR mice (male, *n* = 7)	Reserpine 1 mg/kg for 3 days	FA (5/10/20/40/80 mg/kg) for 30 min	Thermal hyperalgesia, TST, FST
Zhang et al. (2013)	ICR mice (male, *n* = 8)	Reserpine 1 mg/kg for 3 days	FA (5/10/20/40/80 mg/kg) for 2 days	Thermal hyperalgesia, Mechanical allodynia, FST
Lenzi et al. (2015)	Swiss mice (male, *n* = 6–8)	FST, TST	FA (0.01/0.1/1/10 mg/kg/day) for 21 days	FST, TST, OFT
Zeni et al. (2017)	Swiss mice (male, *n* = 6–8)	CORT 20 mg/kg/day for 21 days	FA 1 mg/kg/day for 7 days	FST, TST, OFT, Splash test
Liu et al. (2017)	ICR mice (male, *n* = 10)	CUMS	FA (20/40 mg/kg) for 4 weeks	SPT, FST
Liu et al. (2017b)	ICR mice (male, *n* = 6)	CUMS	FA (20/40 mg/80/kg) for 4 weeks	SPT, TST
Sasaki et al. (2019)	ICR mice (male, *n* = 7)	TST	FA 5 mg/kg/day for 7 days	TST
Zeni et al. (2012)	Swiss mice (male, *n* = 6)	TST	FA 0.01 mg/kg for 15 mins	TST, OFT
Li et al. (2020b)	C57BL/6 mice (male, *n* = 10)	CUS for 21 days	FA (40/80 mg/kg/day) for 14 days	SPT, FST, TST
Deng et al. (2022)	C57BL/6 mice (male, *n* = 10)	LPS	FA 20 mg/kg/day for 7 mins	OFT, EPM, FST, SPT

Abbreviation: CMUS, chronic unpredictable mild stress; CORT, corticosterone; CUS, chronic unpredictable stress; EPM, Elevated Plus‐Maze test; FST, forced swimming test; GI, gastrointestinal; ICR, imprinting control region; LPS, lipopolysaccharide; OFT, open‐field test; SD, Sprague–Dawley; SPT, sucrose preference test; TST, tail suspension test.

**TABLE 3:** Main outcome and mechanism of ferulic acid for depression.

Study (years)	Outcome	Mechanism
Chen et al. (2015)	FA increased monoamine neurotransmitter levels in the hippocampus and frontal cortex	Inhibited MAO‐A activity
	FA increased 5‐HT and norepinephrine in the hypothalamus with higher doses	
	FA decreased the level of 5‐HIAA	
	FA reduced the immobility time in FST and TST	
Zhang et al. (2011a)	FA improved 5‐HT, CRH and ACTH during stress	Regulated HPA axis
	FA selectively inhibited NE and DA reuptakes in brain	
	FA increased 5‐HT in hippocampus	
	FA reduced immobility time, increased locomotor activity, accelerated gastric emptying and intestinal	
Xu et al. (2013)	FA increased 5‐HT, NE and DA levels in the frontal cortex and hippocampus	Improved the monoamine system
	FA reversed the elevation of nitrite and lipid peroxide levels the frontal cortex and hippocampus	Antioxidant activity
	FA increased SOD and GSH levels	Anti‐inflammation
	FA decreased IL‐1β, TNF‐α and substance P levels	Anti‐apoptosis
	FA decreased p65 of NF‐κβ subunit levels and the caspase‐3 activity	
	FA increased in tail flick latency and paw‐withdrawal threshold	
	FA reduced the immobility time in FST and TST	
Zhang et al. (2013)	FA increased thermal and mechanical pain thresholds	Antioxidant activity
	FA increased SOD levels in hippocampus and frontal lobe	
	FA increased the level of 5‐HT and NE	
	FA reduced immobility time in FST	
Lenzi et al. (2015)	FA increased SOD, CAT and GSH‐Px activities in the blood, hippocampus and cerebral cortex	Antioxidant activity
	FA decreased TBA‐RS levels in the blood, hippocampus and cerebral cortex	
	FA reduced the immobility time in FST and TST	
Zeni et al. (2017)	FA decreased MDA, nitrites and PC levels	Antioxidant activity
	FA ameliorated CORT‐induced depressive‐like behavior and nitrosative/oxidative stress	
	FA decreased the immobility time in the TST	
	FA increased the total time of grooming	
Liu et al. (2017)	FA up‐regulated the levels of BDNF, postsynaptic protein PSD95 and synapsin I in the prefrontal cortex and hippocampus	Increased BDNF levels
	FA increased neurotrophin‐related synaptic protein levels	
	FA increased the sucrose preference	
	FA decreased the immobility time	
Liu et al. (2017b)	FA inhibit IL‐1β, IL‐6 and TNF‐α expression in the prefrontal cortex	Anti‐inflammatory
	FA inhibit microglia, NF‐κB signaling and NLRP3 inflammasome activation in the prefrontal cortex	
	FA increased the sucrose preference	
	FA reduced the immobility time	
Sasaki et al. (2019)	FA upregulated energy metabolism‐, cell proliferation‐, and dopaminergic synthesis‐related signaling pathway genes	Enhanced energy metabolism activity
	FA elevated the contents of DA, NE, and BDNF levels in the limbic system of the mice brain	
	FA decreased glycogen level in brain	
	FA increased ATP level	
	FA reduced serum corticosterone level	
	FA Reverses depressive‐like behavior induced by TST	
Zeni et al. (2012)	FA activated PKA, CaMKII, PKC, MAPK/ERK or PI3K signaling pathways	Promoted neuroplasticity, neurogenesis
	FA exerted antidepressant‐like effect in the TST in mice	
Li et al. (2020b)	FA reversed the depression‐like behaviors induced by CUS	Inhibited SIRT6 expression
Deng et al. (2022)	FA effectively alleviated depression‐like behavior of LPS‐induced mice	Manipulated gut microbiome and microbial metabolism
	FA adjusted the gut microbiome distribution in LPS‐induced mice	
	FA regulated metabolomic changed by LPS in mice	

Abbreviation: 5‐HT, serotonin; 5‐HIAA, 5‐hydroxyindoleac etic acid; ACTH, adrenocorticotropic hormone; AKT, protein kinase B; BDNF, brain‐derived neurotrophic factor; CaMKII, calmodulin‐dependent protein kinase II; CAT, catalase; CMUS, chronic unpredictable mild stress; CORT, corticosterone; CRH, corticotropin releasing hormone; CRMP2, collapsin response mediator protein 2; ERK, extracellular signal regulated kinase; GSH, glutathione; GSH‐Px, glutathione peroxidase; IL‐1β, interleukin‐1β; IL‐6, interleukin‐6; LPS, lipopolysaccharide; MAO‐A, monoamine oxidase A; MAPK, mitogen‐activated protein kinase; MDA, malondialdehyde; NE, norepinephrine; PC, protein carbonyl; PI3K, phosphatidylinositol‐3‐kinase; PKA, protein kinase A; TBA‐RS, thiobarbituric acid‐reactive substances; FST, forced swimming test; SIRT6, Sirtuin 6; SOD, superoxide dismutase; TNF‐α, tumor necrosis factor‐α; TST, tail suspension test.

### 5.2 Risk of bias

The risk of bias was associated with studies that ranged from 3 to 5 out of a total of six points. Two studies scored 3 points (16.7%), nine studies scored 4 points (66.7%), and two studies scored 5 points (16.7%). The risk of bias in the 12 studies is shown in Table 4.

**TABLE 4:** Risk of bias of included studies.

Study	Chen et al. (2015)	Liu et al. (2017)	Liu et al. (2017b)	Lenzi et al. (2015)	Sasaki et al. (2019)	Zeni et al. (2012)	Zeni et al. (2017)	Li et al. (2020b)	Xu et al. (2013)	Zhang et al. (2011)	Zhang et al. (2013)	Deng et al. (2022)
A	√	√	√	√	√	√	√	√	√	√	√	√
B	√	√	√	√	√	√	√	√	√	√	√	√
C		√	√	√		√	√	√		√		
D												
E	√	√	√	√	√	√	√	√	√	√	√	√
F					√			√	√	√		√
Total	3	4	4	4	4	4	4	5	4	5	3	4

*Note*: A: peer reviewed publication; B: random allocation to group; C: blinded assessment of outcome; D: a sample size calculation; E: compliance with animal welfare regulations; F: a statement of a potential conflict of interest.

### 5.3 Summary of the studies

The FST and TST were the two main strategies used for assessing depression levels in mice by measuring the duration of immobility among them under desperate conditions. In addition, the sucrose preference test and other tests were used to measure the absence of emotion. Twelve in vivo studies were shown to improve the performance of behavioral tests following FA treatment, suggesting that FA alleviated depression in animal models. Ferulic acid increased the levels of monoamine transmitters in the hippocampus and frontal cortex by inhibiting monoamine oxidase A (MAO‐A) activity in the frontal cortex and hippocampus. Furthermore, it was found that high doses of FA (80 mg/kg) increased the levels of 5‐HT and norepinephrine (NE) in the hypothalamus, and 5‐hydroxyindoleac etic acid (5‐HIAA) is a metabolite of 5‐HT; the reduction of 5‐HIAA level also showed that the level of 5‐HT was improved from another aspect.^71^ Furthermore, Zhang et al.^31^ demonstrated that FA selectively inhibited 5‐HT, NE, and DA reuptakes regulated the HPA axis, increased ghrelin, and stimulated jejunal contraction simultaneously.

Reserpine can affect NE of sympathetic nerve endings and lowers blood pressure and central sedation by depleting NE.^72^ Reserpine has been reported to have several effects on biogenic amines. Furthermore, it was found that oxidative stress may be more conducive to initiating the onset of depression.^73^ Xu et al. administered reserpine to ICR mice for three consecutive days to construct a depression model. After intervention with reserpine, neurotransmitters (5‐HT, 5‐HIAA, NE, DA) in the brain of mice decreased, while oxidative stress, inflammatory factors, and indicators of apoptosis increased. Behavioral tests for heat hyperalgesia, TST, and FST were performed 30 minutes after treatment with FA. The results showed that FA increased tail‐flick latency and paw‐withdrawal threshold. Furthermore, FA reduced the immobility time in FST and TST. Moreover, FA increased the 5‐HT, NE, and DA levels in the frontal cortex and hippocampus via regulating the monoamine system. While FA decreased the level of interleukin‐1β (IL‐1β), tumor necrosis factor‐α (TNF‐α), and substance P to exert anti‐inflammatory effects. FA also decreased the level of nitrite and lipid peroxide and increased superoxide dismutase (SOD) and glutathione peroxidase (GSH) levels to exert an anti‐oxidative stress effect. It was found that FA inhibited caspase‐3 levels both in the frontal cortex and hippocampus of the mice treated with reserpine. These results suggest that FA exerted antidepressant effects by modulating the monoaminergic system, anti‐inflammatory, anti‐oxidative stress, and anti‐apoptotic mechanisms.^74^ Similarly, Zhang et al.^75^ reached a consistent conclusion in the same animal model, confirming that the treatment of FA can increase the thermal and mechanical pain thresholds of ICR mice.

Furthermore, two additional studies concluded that FA can improve depression in mice through an antioxidant pathway. Lenzi et al.^76^ pointed out that FA increased SOD, catalase (CAT), and glutathione peroxidase (GSH‐Px) activities, and decreased thiobarbituric acid‐reactive substances (TBA‐RS) levels in the blood, hippocampus, and cerebral cortex. Zeni et al.^77^ found that FA decreased malondialdehyde (MDA), nitrites, and Protein carbonyl (PC) levels; thus, FA could improve various behavioral disorders.

Owing to chronic stress, FA can induce behavioral and biochemical changes in mice and cause inflammation of the nervous system and dysfunction of neurological function.^78^ The long‐term stress exacerbates the process of depression. In the chronic unpredictable mild stress (CUMS) mice model, FA increased the sucrose preference and decreased the immobility time after 4 weeks of treatment through the mechanism of upregulating the levels of BDNF, postsynaptic protein postsynaptic density 95 (PSD95), and synapsin I in the prefrontal cortex and hippocampus. This suggests that FA exerts the antidepressant‐like effects on behaviors by increasing neurotrophin‐related synaptic protein levels.^79^ Meanwhile, Liu et al.^80^ also used CUMS‐induced ICR mice models and found that FA downregulated inflammatory factors such as IL‐1β, IL‐6, and TNF‐α after 4 weeks of treatment. In addition, FA inhibited microglia, NF‐κB signaling, and NLRP3 inflammasome activation in the prefrontal cortex. Ferulic acid improved depression in mice through an anti‐inflammatory approach. A growing body of studies suggested that overgrowth of detrimental gut microbes is driving the development of depression.^81^ Ferulic acid effectively regulated gut microbiome and microbial metabolism in a variety of disease settings, including neuroinflammation.^82^ Deng et al.^31^, ^83^ demonstrated that FA effectively alleviated depression‐like behavior in mice, through increasing *Solibacillus*, *Firmicutes*, *Arthrobacter*, and *Acinetobacter* abundance, decreasing *Oscollospira*, *Parabacteroides*, and *Rummeliibacillus* abundance, as well as elevating ghrelin and evoking the jejunal contraction activity, which indicated that changed gut microbiome structure and microbial metabolism were another therapeutic mechanism of FA in treating depression.

Dysfunctional mitochondria can cause abnormal energy metabolism and accelerate the onset of depression. Ferulic acid can promote glycogen metabolism in the brain and increase ATP levels, which is beneficial for elevating the contents of DA, NE, and BDNF levels in the limbic system. FA‐enhanced energy production is one of the underlying mechanisms of the antidepressive effects of FA.^84^ Furthermore, FA activated the signaling pathways (PKA, CaMKII, PKC, MAPK/ERK, or PI3K) related to neuroplasticity, neurogenesis, and cell survival to improve depressive symptoms in mice.^85^ SIRT6 and its downstream AKT/CRMP2 signaling pathway may also be one of the therapeutic mechanisms that FA uses to alleviate depressive behavior. Moreover, FA regulated AKT/CRMP2 pathway by inhibiting SIRT6 expression, and ameliorated CUS‐induced depression‐like behaviors in mice.^86^

## 6 DISCUSSION

A total of 100 animals from 12 preclinical trials were included in the analysis. The results showed that FA improved depression‐related behavioral abnormalities and restored 5‐HT, DA, and NE levels compared with the control group. This suggests that FA plays a therapeutic role in animal depression models through regulating the monoaminergic system, inhibiting HPA axis overactivation, antioxidant, anti‐inflammatory, and anti‐apoptotic mechanisms. Based on current knowledge, FA is undoubtedly beneficial in the treatment of depression in animal models. However, it is still unclear whether animal studies can serve as reliable guides for human studies. Further evidence is required in this regard to assess FA in clinical trials.

**FIGURE 3:**
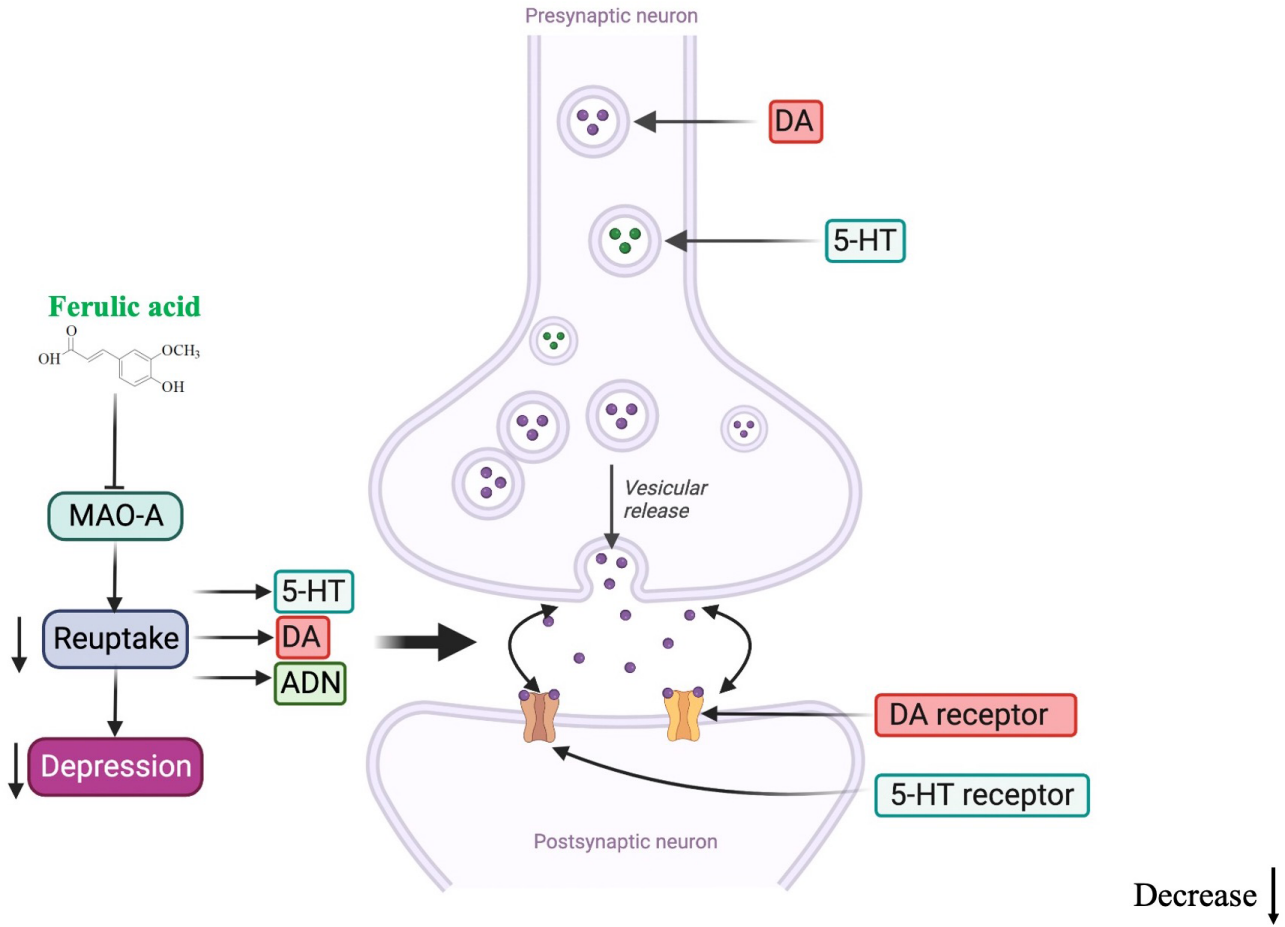
Regulation of monoaminergic neurotransmission. Ferulic acid increases the 5‐HT, ADN, and DA levels in the synaptic cleft of frontal cortex and hippocampus via regulating the monoamine system. FA, ferulic acid; DA, dopamine; 5‐HT, serotonin; AND, adrenaline.

Impairment of monoaminergic neurotransmission and concomitant reductions in extracellular concentrations of 5‐HT and NE are the main causes of depression and the target of most antidepressants.^87^ Currently, most antidepressants work by inhibiting monoamine reuptake, increasing monoamine levels in the synaptic cleft.^88^ However, selective serotonin reuptake inhibitors may cause withdrawal symptoms such as nausea, vomiting, and diarrhea, thus limiting their clinical application.^89^ Ferulic acid was found to increase the levels of monoamine neurotransmitters in the hippocampus and frontal cortex of the mouse brain. Furthermore, the 5‐HT and NE increased significantly in the hypothalamus by 40–80 mg/kg after treatment with FA. Furthermore, the 5‐HIAA/5‐HT ratio decreased in the hippocampus and frontal cortex of mice after FA treatment, suggesting a reduction in neurotransmitter metabolism in the CNS. MAO‐A is involved in the metabolism of 5‐HT and NE, whereas FA may play a role in regulating the expression of 5‐HT and NE by inhibiting the activity of MAO‐A in the frontal cortex and hippocampus.^71^ Similarly, other studies have reached the same conclusion. Zeni et al.^85^ found that FA (0.01 mg/kg, p.o.) exerted antidepressant effects by inhibiting 5‐HT reuptake and modulating 5‐HT1A and 5‐HT2 receptors. Furthermore, FA may exert effects similar to tricyclic antidepressants, thus reducing symptoms of depression by selectively inhibiting 5‐HT, NE, and uptaking DA in the brain^31^ (Figure 3).

**FIGURE 4:**
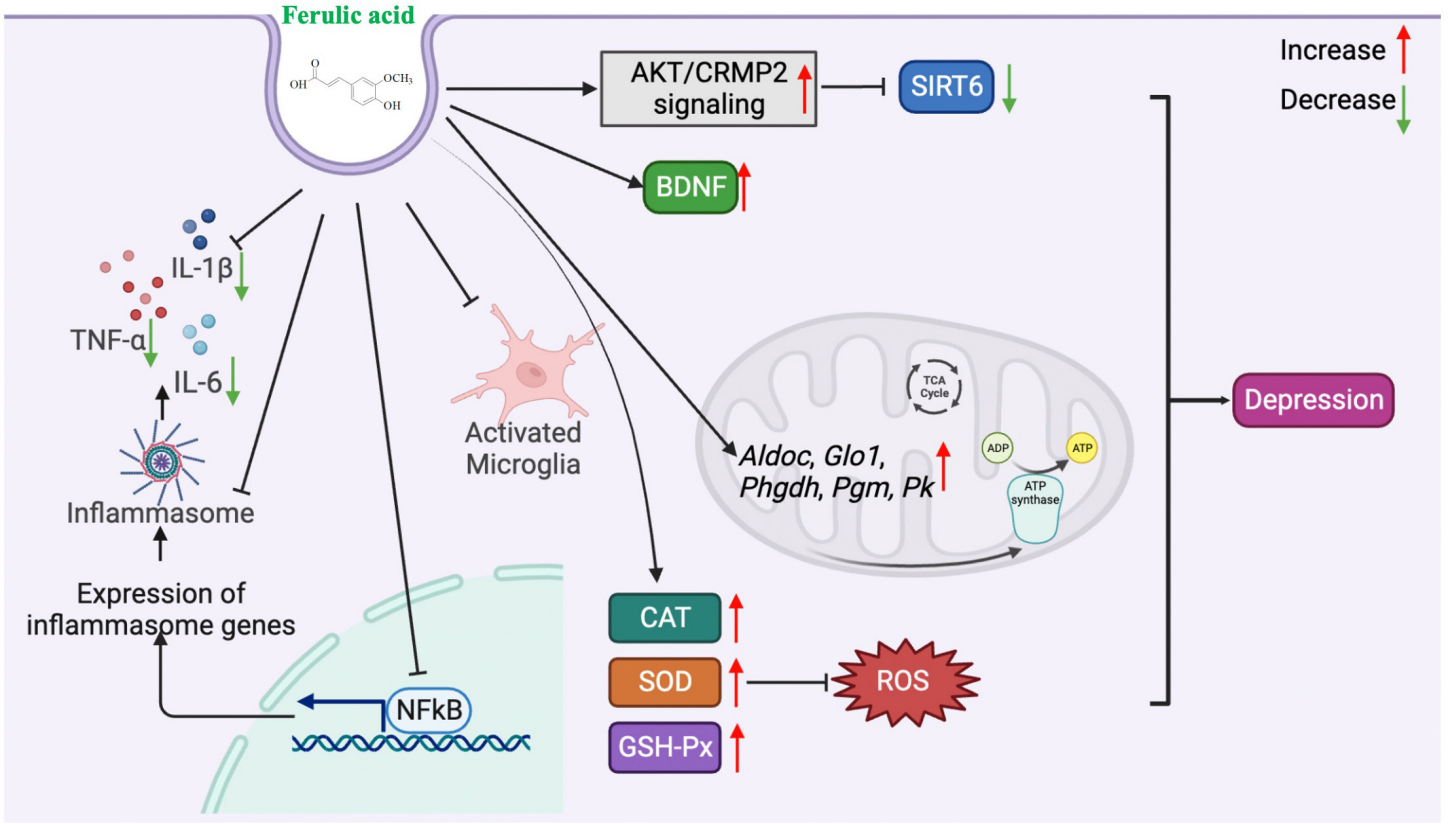
Multipathway antidepressant mechanisms of FA. FA inhibits microglia, NF‐κB signaling, NLRP3 inflammasome activation, IL‐1β, IL‐6, and TNF‐α expression; FA can also increase CAT, SOD, and GSH‐Px activities in the blood, hippocampus, and cerebral cortex of mice, as well as inhibit ROS production; FA treatment increases BDNF levels in prefrontal cortex and hippocampus to serve anti‐depressives effect; FA promotes the expression of glycolytic genes (*Aldoc*, *Glo1*, *Phgdh*, *Pgm*, and *Pk*), as well as mitochondrial tricarboxylic acid cycle; FA inhibits SIRT6 expression through stimulating AKT/CRMP2 signaling pathways. AKT, protein kinase B; BDNF, brain‐derived neurotrophic factor; CAT, catalase; FA, ferulic acid; GSH‐Px, glutathione peroxidase; IL, interleukin; NF‐κB, nuclear factor‐kappa B; NLRP3, NOD‐like receptor family pyrin domain containing 3; ROS, reactive oxygen species; SOD, superoxide dismutase.

Neuroinflammation also contributes to the pathogenesis of depression. Pro‐inflammatory cytokines such as IL‐1β, IL‐6, and TNF‐α are expressed highly in the plasma or brain of depression‐like animals.^90^ Activated microglia, which are a major source of pro‐inflammatory cytokines in the brain, release multiple pro‐inflammatory cytokines, induce neuroinflammation, and exacerbate neuronal death.^91^ Microglia, NF‐κB signaling, NLRP3 inflammasome activation, IL‐1β, IL‐6, and TNF‐α expression were significantly elevated in the prefrontal cortex of the mice in the CUMS depression model. However, these inflammatory responses induced by CUMS were reversed by FA, suggesting that the antidepressive effects of FA may be related to anti‐inflammation^80^ (Figure 4).

**FIGURE 5:**
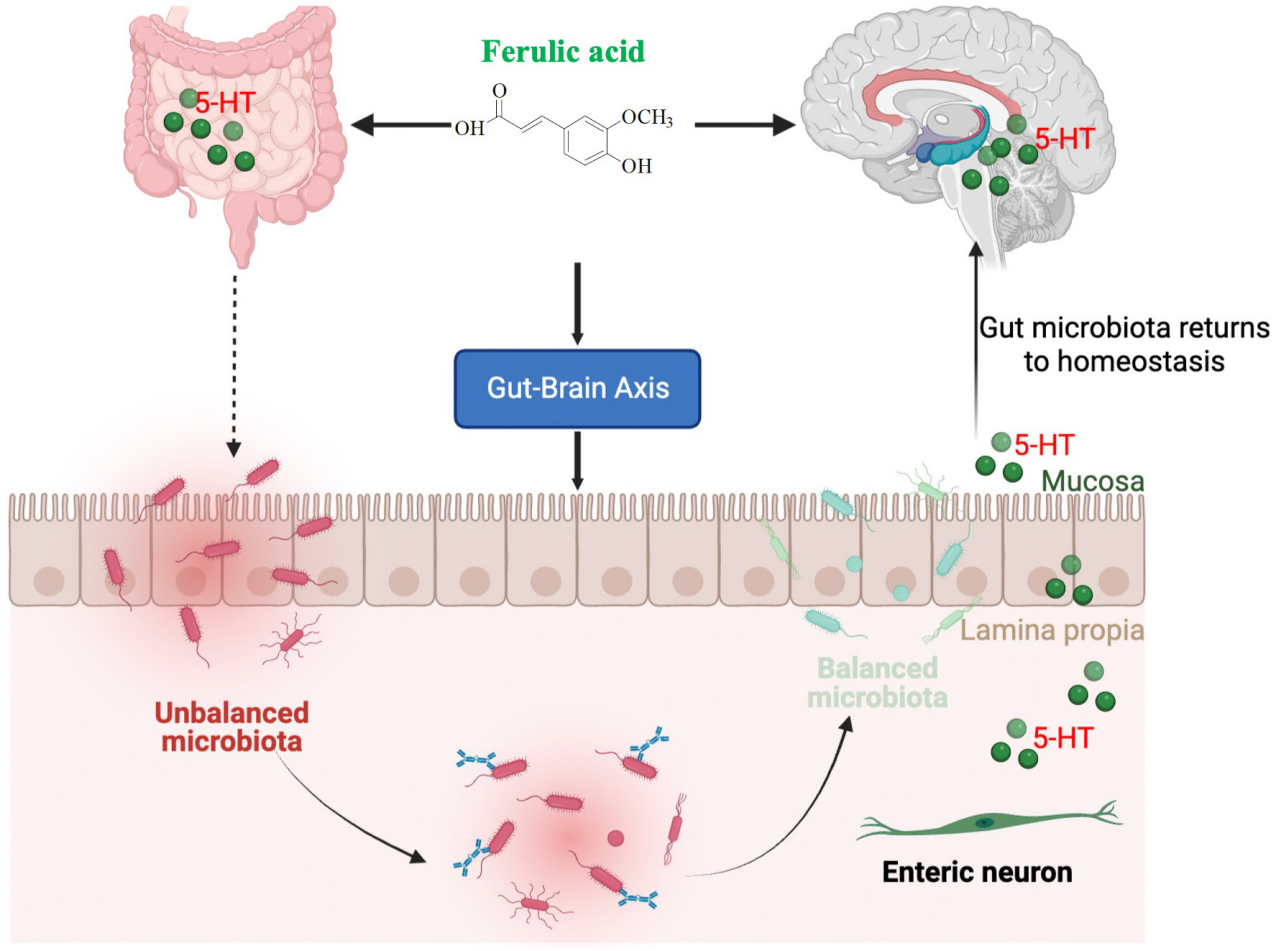
Regulative role of FA in gut–brain axis. Ferulic acid modulates gut microbiota diversity and subsequently involved in gut–brain axis; FA is related with the 5‐HT system and ultimately contributes to the regulation between gut homeostasis and brain function. 5‐HT, 5‐hydroxytryptamine; FA, ferulic acid.

The imbalance of intestinal flora can trigger systemic inflammatory responses, including neuroinflammation, which lays a foundation for us to target intestinal flora to prevent depression.^64^ *Firmicutes*, *Proteobacteria*, and *Bacteroidetes* are the main phylum of intestinal flora. *Firmicutes* are associated with depression in mice.^92^ Previous studies have demonstrated that the ratio of *Firmicutes* to *Bacteroidetes* can be used as a parameter to evaluate the level of inflammation in mice. The lower the ratio, the worse the inflammation.^93^ Ferulic acid restored *Firmicutes* abundance, attenuated the decreased value of *Firmicutes* to *Bacteroidetes* ratio, and the *Prevotella*, *Parabacteroides*, and *Oscillospira* abundance, as well as salvaged the abundance of *Acinetobacter* induced by lipopolysaccharide.^83^ Additionally, the promotion of jejunal contraction and intestinal movement by increasing ghrelin was also one of the important mechanisms of FA in treating depression^31^ (Figure 5).

Oxidative stress is also involved in the onset of depression, which may be associated with the increase of ROS and the loss of the antioxidant defense system.^94^ The levels of antioxidant enzymes (CAT, SOD, GSH‐Px) decreased, and those of ROS increased in the serum of the patients with depression.^95^ Previous studies confirm the antioxidative properties of FA.^96^ In the TST and FST stress‐induced mice, the despair test reduced CAT, SOD, and GSH‐Px activity in the blood and cerebral cortex of mice. Furthermore, the test increased the level of TBA‐RS when evaluating the regulation of the antioxidant system. Ferulic acid had antidepressant effects without affecting motor activity at the dose of 1 mg/kg. FA can also increase CAT, SOD, and GSH‐Px activities in the blood, hippocampus, and cerebral cortex of mice, while decreasing levels of TBA‐RS. These findings demonstrate that low doses of FA can play an antidepressant role when the antioxidant defense system is modulated.^76^ Corticosterone treatment has been used as evidence to develop a depression‐like state in animals, which mimics the HPA‐axis dysregulation involved in the development of depression.^97^ Furthermore, CORT increases levels of TBA‐RS, PC, and nitrite in the brain, whereas it decreases levels of nonprotein sulfhydryl groups. FA (1 mg/kg) decreased TBA‐RS, PC, and nitrite levels, while it increased NPSH levels, and improved depressive‐like behavior. These results indicate that FA is a glutamate antagonist and antioxidant compound that can improve the oxidative stress induced by corticosterone.^77^ In a mouse model of reserpine‐induced pain and depression, lipid peroxide (LPO) and nitrite levels were significantly upregulated, whereas SOD and GSH levels were downregulated in the frontal cortex and hippocampus, indicating the activation of oxidative and nitrification stress. FA (40 or 80 mg/kg, p.o.) reversed reserpine‐induced behavioral abnormalities and increased 5‐HT, NE, and DA levels in the hippocampus and frontal cortex. Meanwhile, FA can effectively resist oxidative stress and neuroinflammation, downregulate LPO, IL‐1β and TNF‐α levels, as well as upregulate GSH and SOD levels. Furthermore, FA reduced the levels of substance P, NF‐κB, and caspase‐3 in a dose‐dependent manner in the frontal cortex and hippocampus of reserpine mice. These results suggested that FA played a therapeutic role in reserpine‐induced pain and depression‐like behavior by regulating the monoaminergic system, anti‐inflammatory, and anti‐apoptotic signaling pathways and inhibiting oxidative stress.^74^, ^75^

The neurotrophic hypothesis suggests that decreased NTF trigger neuronal atrophy, whereas decreased hippocampal synapse protein synthesis and neurogenesis trigger depression.^98^ Antidepressants have been reported to improve this neurotrophic factor deficit, thus reversing cell loss.^99^ Recent clinical studies reported that BDNF levels in the peripheral blood of patients with depression reduced indirectly reflected biochemical changes in the brain.^100^, ^101^ Effective antidepressant treatments such as antidepressants, sleep deprivation, and electroconvulsive therapy can directly increase BDNF levels in depressed patients.^102^, ^103^, ^104^ The chronic unpredictable mild stress is a prevalent depression‐like model that can induce a variety of behavioral and biochemical changes among animals^105^ and has been shown to significantly reduce BDNF levels in mice's prefrontal cortex and hippocampus. Chronic FA treatment reversed this deficit, suggesting that BDNF is involved in the antidepressives effect of FA.^79^

Similarly, in a TST‐induced depressive mice model, Sasaki et al. found that FA significantly increased the levels of BDNF protein expression in mice brains. We highlight that FA potentially regulates mitochondrial function. Early mitochondrial dysfunction is a symptom of major depressive disorder among adult patients. Furthermore, evidence suggests that mitochondrial function and energy metabolism have important effects on social behavior.^106^ In the mouse model, FA treatment increased the ATP level in the limbic area of the brain. Moreover, FA promoted the expression of glycolytic genes (*Aldoc*, *Glo1*, *Phgdh*, *Pgm*, and *Pk*), as well as mitochondrial tricarboxylic acid cycle and electron transport chain genes (*IDH3a*, *Ndufab*, *Pdk4*, *Sdha*, and *Uqcrc1*) in mice brains. Therefore, increasing ATP production and activating gene expression related to energy metabolism may also contribute to the antidepressive mechanisms of FA.^84^

Sirtuins (SIRTs) are a family of nicotinamide adenine dinucleotide (NAD+)‐dependent deacetylases that regulate many cellular physiological processes.^107^ More recently, SIRT has been associated with mood disorders in mice and humans.^108^ Li et al.^109^ showed that hippocampal SIRT6 expression was significantly upregulated in the CUS‐induced mouse model. The deregulation of the hippocampal SIRT6 could strongly prevent CUS‐induced depression‐like phenotypes. Increasing evidence indicates that the PI3K/AKT signaling is involved in the mediating antidepressant effects. Pharmacological inhibition of SIRT6 is robust and effective at preventing depressive behaviors through stimulating AKT/CRMP2 signaling pathways. Specifically, FA inhibited SIRT6 expression dose‐dependently and improved CUS‐induced depression‐like behaviors. Moreover, FA played a neuroprotective effect through the SIRT6/AKT/CRMP2 pathway.^86^

**FIGURE 6:**
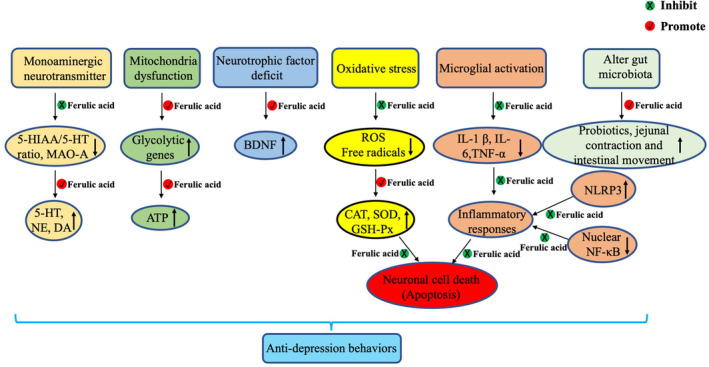
Schematic representation of the neuroprotective mechanisms of ferulic acid in treating depression. Ferulic acid was shown to improve behavioral disorders of depression and exhibited regulation of monoaminergic neurotransmission, anti‐inflammation, anti‐oxidation, and altered gut microbiota among other mechanisms. Ferulic acid also serves as a neuroprotective agent used in depression. Red words indicate the increased expression and green words indicate the decreased expression.

Indeed, the antidepressant and prokinetic effects of FA simultaneously highlight the multiple pharmacology of its antidepressant action. Ferulic acid significantly increases 5‐HT in hippocampus, suggesting that FA selectively inhibited 5‐HT reuptake in brain. FA acts on the HPA axis, increasing CRH, ACTH, and 5‐HT levels during stress. Additionally, FA alleviates depression by selectively inhibiting NE and DA reuptake. Moreover, FA promotes intestinal motility by increasing hippocampal and plasma ghrelin levels, which is also supported by FA‐induced jejunal contraction. Ferulic acid improving the composition of the intestinal flora of patients with depression will become a cutting‐edge treatment method for depression, making it possible for personalized and precise treatment of depression (Figure 6). However, to determine the exact clinical efficacy of FA‐regulated probiotics in the treatment of depression, further large‐scale population trials and follow‐up data are required for the persistence of antidepressant effects.

## 7 CONCLUSION

Phytomedicines have gained global recognition for their extensive practical experience and positive therapeutic effect in the prevention and treatment of diseases. Recently, several studies have shown that FA is a natural protective agent that requires further development. Ferulic acid exerts important neuroprotective effects in CNS diseases through antioxidant, anti‐inflammatory, anti‐apoptosis, alter gut microbiota and other mechanisms.^110^, ^111^ Moreover, FA was found to alleviate abnormal depressive behaviors in multiple animal depression models, underscoring the potential use of FA as a new antidepressant agent. However, the dosage and administration time of FA in animal models is not consistent, small number of animal models were included and no pharmacokinetic studies of FA in animal models of depression have been performed. Furthermore, there is a lack of evidence that supports the direct translation of animal trials into human trials. Therefore, future research should focus on how to accelerate the transformation of the antidepressant effect of FA in human trials and improve the design of randomized controlled clinical trials to ensure the clinical application of FA among patients with depression.

## AUTHOR CONTRIBUTIONS

Xiaoyu Dong was involved in data curation, formal analysis, investigation, methodology, and writing—original draft. Dongxue Zhao was involved in conceptualization, supervision, validation, and writing—review and editing.

## CONFLICT OF INTEREST STATEMENT

The authors declare that they have no competing interests.

## Data Availability

Data sharing not applicable to this article as no datasets were generated or analyzed during this study.
